# Carbon­yl(η^5^-cyclo­penta­dien­yl)(pyridine)(triethyl­stann­yl)iron(II)

**DOI:** 10.1107/S1600536808037781

**Published:** 2008-11-20

**Authors:** Masumi Itazaki, Masahiro Kamitani, Hiroshi Nakazawa

**Affiliations:** a Department of Chemistry, Graduate School of Science, Osaka City University, Sumiyoshi-ku, Osaka 558-8585, Japan

## Abstract

In the title complex, [Fe(C_5_H_5_){Sn(C_2_H_5_)_3_}(C_5_H_5_N)(CO)], the Fe atom is coordinated by carbonyl, pyridine, triethyl­stannyl and cyclo­penta­dienyl ligands in a typical three-legged piano-stool configuration. The Fe—Sn and Fe—N bond distances are 2.5455 (13) and 1.984 (6) Å, respectively.

## Related literature

For background, see: Nakazawa *et al.* (2007 ▶). Applications of transition metal complexes with a stannyl ligand are reviewed by Smith *et al.* (2000 ▶). For a related transition metal stannyl complex having a pyridine ligand, see: Rickard *et al.* (1999 ▶). For structures of related silyl analogues, see: Iwata *et al.* (2003 ▶); Nakazawa *et al.* (2007 ▶); Itazaki *et al.* (2007 ▶).
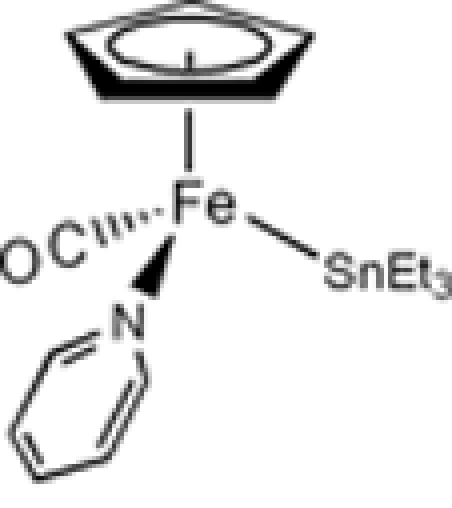


## Experimental

### 

#### Crystal data

[FeSn(C_2_H_5_)_3_(C_5_H_5_)(C_5_H_5_N)(CO)]
*M* *_r_* = 433.92
Monoclinic, 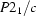

*a* = 15.038 (4) Å
*b* = 7.8271 (19) Å
*c* = 15.717 (4) Å
β = 96.783 (5)°
*V* = 1837.1 (8) Å^3^
*Z* = 4
Mo *K*α radiation
μ = 2.15 mm^−1^
*T* = 200 (2) K
0.13 × 0.13 × 0.02 mm

#### Data collection

Rigaku/MSC Mercury CCD diffractometer
Absorption correction: multi-scan (Jacobson, 1998 ▶) *T*_min_ = 0.768, *T*_max_ = 0.958
17586 measured reflections
4174 independent reflections
3439 reflections with *I* > 2σ(*I*)
*R*_int_ = 0.069

#### Refinement

*R*[*F* ^2^ > 2σ(*F* ^2^)] = 0.088
*wR*(*F* ^2^) = 0.138
*S* = 1.07
4174 reflections
193 parameters
3 restraints
H-atom parameters constrained
Δρ_max_ = 0.54 e Å^−3^
Δρ_min_ = −0.50 e Å^−3^

### 

Data collection: *CrystalClear* (Rigaku, 2001 ▶); cell refinement: *CrystalClear*; data reduction: *CrystalClear*; program(s) used to solve structure: *SHELXS97* (Sheldrick, 2008 ▶); program(s) used to refine structure: *SHELXL97* (Sheldrick, 2008 ▶); molecular graphics: *ORTEP-3 for Windows* (Farrugia, 1997 ▶); software used to prepare material for publication: *SHELXL97*.

## Supplementary Material

Crystal structure: contains datablocks I, FePySnR3. DOI: 10.1107/S1600536808037781/wm2203sup1.cif

Structure factors: contains datablocks I. DOI: 10.1107/S1600536808037781/wm2203Isup2.hkl

Additional supplementary materials:  crystallographic information; 3D view; checkCIF report

## Figures and Tables

**Fig. 1.**
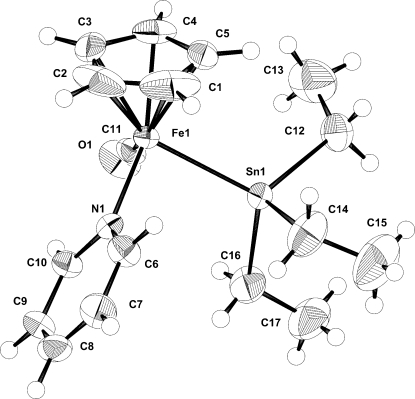
The molecule of [(CO)(C5H5)(Sn(C2H5)3)(C5H5N)Fe], (I), showing the atom-labelling scheme. Displacement ellipsoids are drawn at the 50% probability level. H atoms have been omitted for clarity.

## supplementary crystallographic information

### Comment

Transition metal complexes with a stannyl ligand have attracted considerable attention because they are very important intermediates in hydrostannation reaction (Smith *et al.*, 2000). On the other hand, we reported that an iron complex with a pyridine ligand acts as a good precursor in the C—CN bond cleavage of organonitriles (Nakazawa *et al.*, 2007). A large number of stannyl complexes and of pyridine complexes have been synthesized, however only one crystal structure has been reported to date for a transition metal stannyl complex having a pyridine ligand, *viz.* [Os(CO)(Me)(PPh_3_)_2_(SnMe_2_Cl)(py)] (Rickard *et al.*, 1999). This paper is the first report of the molecular and crystal structure of an Fe—Sn complex with pyridine.

The title complex, [(CO)(C_5_H_5_)(Sn(C_2_H_5_)_3_)(C_5_H_5_N)Fe], (I), was synthesized by the reaction of (C_5_H_5_)(CO)_2_Fe(SnEt_3_) with pyridine under photolytic conditions. An *ORTEP* drawing of the molecule is displayed in Fig. 1. Complex (I) has a typical three–legged piano–stool configuration: the Fe atom has a terminal CO ligand, a triethylstannyl ligand, a pyridine and a cyclopentadienyl ligand (Cp) with the latter bonded in an η^5^–fashion. The Fe1—Sn1, Fe1—N1 and Fe1—C11 distances [2.5455 (13), 1.984 (6), 1.728 (9) Å] in (I) are comparable to the silyl analogues; [Cp^*^(CO)Fe(SiMe_2_NPh_2_)(py)] [Cp^*^ = C_5_Me_5_, 2.3330 (4), 1.991 (1), 1.716 (2) Å; Iwata *et al.*, 2003], [(C_5_H_5_)(CO)Fe(SiEt_3_)(py)] [2.303 (5), 1.9823 (17), 1.719 (2) Å; Nakazawa *et al.*, 2007], and [(C_5_H_5_)(CO)Fe(SiEt_3_)(C_5_H_5_N-3,5-CH_3_)] [2.3341 (9), 1.982 (2), 1.722 (3) Å; Itazaki *et al.*, 2007]. The N1—Fe1—Sn1 angle [88.59 (19)°] is slightly narrower than that for [(C_5_H_5_)(CO)Fe(SiEt_3_)(py)] [89.25 (10)°; Nakazawa *et al.*, 2007].

### Experimental

A benzene solution (8 ml) containing (C_5_H_5_)(CO)_2_Fe(SnEt_3_) (0.98 mmol, 374 mg) and pyridine (4.88 mmol, 0.40 ml) was photoirradiated with a 400 W medium pressure mercury arc lamp at 298 K for several hours in nitrogen atmosphere. During the irradiation, the generated CO was degassed several times. Removing volatile materials under reduced pressure led to the formation of a dark red oil, which was dissolved in hexane (1 ml). After the hexane solution was cooled at 233 K for 3 h, the resulting dark red crystals were filtered off and dried *in vacuo* to give (C_5_H_5_)(CO)Fe(SnEt_3_)(py) (I) (0.94 mmol, 407 mg, 96% yield). Spectroscopic analysis: ^1^H NMR (400 MHz, C_6_D_6_, δ, p.p.m.): 1.10 (q, *J*_HH_ = 8.5 Hz, 6H, Sn(C*H*_2_CH_3_)), 1.41 (t, *J*_HH_ = 8.5 Hz, 9H, Sn(CH_2_C*H*_3_)), 4.19 (s, 5H, C_5_H_5_), 5.83 (t, *J*_HH_ = 6.1 Hz, 2H, py), 6.38 (t, *J*_HH_ = 6.1 Hz, 1H, py), 8.55 (d, *J*_HH_ = 6.1 Hz, 2H, py). ^13^C{^1^H} NMR (100.4 MHz, C_6_D_6_, δ, p.p.m.): 3.46 (s, *J*_CSn_ = 78.8 Hz, Sn(*C*H_2_CH_3_)), 12.70 (s, *J*_CSn_ = 10.0 Hz, Sn(CH_2_*C*H_3_)), 79.05 (s, Cp), 122.85 (s, py), 133.77 (s, py), 157.91 (s, py), 223.58 (s, CO). ^119^Sn{^1^H} NMR (149.2 MHz, C_6_D_6_, δ, p.p.m.): 128.32. Anal. Calc. for C_17_H_25_NOSnFe: C, 47.05; H, 5.81; N, 3.23. Found: C, 46.50; H, 5.72; N, 3.09%.

### Refinement

All H atoms were positioned geometrically and treated using a riding model, with C—H distances assumed to be 0.99 Å for methylene H atoms, 0.98 Å for methyl H atoms, and 0.95 Å for aromatic H atoms. The *U*_iso_(H) values were taken to be 1.2*U*_eq_(C) of the respective parent carbon atom.

### Crystal data

**Table:**

[FeSn(C_2_H_5_)_3_(C_5_H_5_)(C_5_H_5_N)(CO)]	*F*_000_ = 872
*M**_r_* = 433.92	*D*_x_ = 1.569 Mg m^−^^3^
Monoclinic, *P*2_1_/*c*	Mo *K*α radiation λ = 0.71070 Å
Hall symbol: -P 2ybc	Cell parameters from 4766 reflections
*a* = 15.038 (4) Å	θ = 4.1–27.5º
*b* = 7.8271 (19) Å	µ = 2.15 mm^−^^1^
*c* = 15.717 (4) Å	*T* = 200 (2) K
β = 96.783 (5)º	Platelet, red
*V* = 1837.1 (8) Å^3^	0.13 × 0.13 × 0.02 mm
*Z* = 4	

### Data collection

**Table:**

Rigaku/MSC Mercury CCD diffractometer	3439 reflections with *I* > 2σ(*I*)
Detector resolution: 14.6199 pixels mm^-1^	*R*_int_ = 0.069
ω scans	θ_max_ = 27.5º
Absorption correction: multi-scan(Jacobson, 1998)	θ_min_ = 4.1º
*T*_min_ = 0.768, *T*_max_ = 0.958	*h* = −19→18
17586 measured reflections	*k* = −10→8
4174 independent reflections	*l* = −20→19

### Refinement

**Table:**

Refinement on *F*^2^	3 restraints
Least-squares matrix: full	H-atom parameters constrained
*R*[*F*^2^ > 2σ(*F*^2^)] = 0.088	*w* = 1/[σ^2^(*F*_o_^2^) + 25.1156*P*] where *P* = (*F*_o_^2^ + 2*F*_c_^2^)/3
*wR*(*F*^2^) = 0.138	(Δ/σ)_max_ < 0.001
*S* = 1.07	Δρ_max_ = 0.54 e Å^−^^3^
4174 reflections	Δρ_min_ = −0.49 e Å^−^^3^
193 parameters	Extinction correction: none

### Special details

**Table:**

Geometry. All e.s.d.'s (except the e.s.d. in the dihedral angle between two l.s. planes) are estimated using the full covariance matrix. The cell e.s.d.'s are taken into account individually in the estimation of e.s.d.'s in distances, angles and torsion angles; correlations between e.s.d.'s in cell parameters are only used when they are defined by crystal symmetry. An approximate (isotropic) treatment of cell e.s.d.'s is used for estimating e.s.d.'s involving l.s. planes.
Refinement. Refinement of *F*^2^ against ALL reflections. The weighted *R*-factor *wR* and goodness of fit *S* are based on *F*^2^, conventional *R*-factors *R* are based on *F*, with *F* set to zero for negative *F*^2^. The threshold expression of *F*^2^ > σ(*F*^2^) is used only for calculating *R*-factors(gt) *etc*. and is not relevant to the choice of reflections for refinement. *R*-factors based on *F*^2^ are statistically about twice as large as those based on *F*, and *R*- factors based on ALL data will be even larger.

### Fractional atomic coordinates and isotropic or equivalent isotropic displacement parameters (Å^2^)

**Table:**

	*x*	*y*	*z*	*U*_iso_*/*U*_eq_	
Fe1	0.17373 (7)	0.08483 (15)	0.47425 (7)	0.0262 (3)	
C1	0.2299 (11)	−0.1470 (15)	0.5222 (7)	0.078 (4)	
H1A	0.2698	−0.2160	0.4944	0.093*	
C2	0.1358 (12)	−0.1582 (16)	0.5128 (7)	0.086 (4)	
H2A	0.1004	−0.2352	0.4764	0.103*	
C3	0.1044 (7)	−0.0394 (19)	0.5652 (7)	0.070 (3)	
H3A	0.0433	−0.0214	0.5725	0.084*	
C4	0.1768 (8)	0.0509 (14)	0.6059 (6)	0.052 (3)	
H4A	0.1738	0.1426	0.6451	0.062*	
C5	0.2532 (7)	−0.0163 (15)	0.5796 (6)	0.054 (3)	
H5A	0.3125	0.0210	0.5978	0.065*	
N1	0.1412 (4)	0.0387 (8)	0.3504 (4)	0.0274 (14)	
C6	0.1798 (6)	−0.0873 (12)	0.3099 (5)	0.040 (2)	
H6A	0.2255	−0.1524	0.3419	0.048*	
C7	0.1574 (7)	−0.1279 (13)	0.2252 (6)	0.049 (2)	
H7A	0.1872	−0.2181	0.1996	0.059*	
C8	0.0916 (7)	−0.0367 (13)	0.1782 (5)	0.045 (2)	
H8A	0.0745	−0.0626	0.1195	0.054*	
C9	0.0508 (6)	0.0922 (13)	0.2171 (5)	0.042 (2)	
H9A	0.0048	0.1577	0.1858	0.050*	
C10	0.0771 (6)	0.1266 (11)	0.3024 (5)	0.0334 (19)	
H10A	0.0483	0.2173	0.3285	0.040*	
C11	0.1287 (6)	0.2880 (12)	0.4731 (5)	0.035 (2)	
O1	0.0971 (5)	0.4230 (9)	0.4738 (4)	0.0531 (18)	
Sn1	0.31362 (4)	0.23728 (8)	0.43713 (3)	0.03269 (17)	
C12	0.3938 (8)	0.3606 (18)	0.5443 (7)	0.072 (4)	
H12A	0.4362	0.2750	0.5719	0.086*	
H12B	0.4297	0.4514	0.5209	0.086*	
C13	0.3452 (9)	0.4377 (17)	0.6116 (8)	0.083 (4)	
H13A	0.2973	0.5118	0.5848	0.107*	
H13B	0.3870	0.5054	0.6506	0.107*	
H13C	0.3193	0.3470	0.6438	0.107*	
C14	0.4046 (8)	0.0648 (16)	0.3820 (8)	0.071 (3)	
H14A	0.4156	−0.0345	0.4208	0.086*	
H14B	0.3739	0.0217	0.3270	0.086*	
C15	0.4914 (9)	0.134 (2)	0.3659 (11)	0.108 (5)	
H15A	0.4826	0.2192	0.3202	0.141*	
H15B	0.5291	0.0410	0.3485	0.141*	
H15C	0.5206	0.1871	0.4183	0.141*	
C16	0.2821 (7)	0.4348 (14)	0.3418 (7)	0.061 (3)	
H16A	0.2572	0.3782	0.2878	0.073*	
H16B	0.2338	0.5063	0.3608	0.073*	
C17	0.3541 (8)	0.5497 (17)	0.3217 (8)	0.081 (4)	
H17A	0.3804	0.6069	0.3742	0.105*	
H17B	0.3295	0.6355	0.2800	0.105*	
H17C	0.4004	0.4833	0.2975	0.105*	

### Atomic displacement parameters (Å^2^)

**Table:**

	*U*^11^	*U*^22^	*U*^33^	*U*^12^	*U*^13^	*U*^23^
Fe1	0.0280 (6)	0.0289 (6)	0.0216 (5)	−0.0011 (5)	0.0021 (4)	0.0025 (5)
C1	0.136 (9)	0.048 (7)	0.053 (7)	0.046 (7)	0.027 (7)	0.031 (6)
C2	0.150 (10)	0.053 (7)	0.044 (6)	−0.052 (8)	−0.029 (7)	0.025 (5)
C3	0.039 (5)	0.113 (10)	0.055 (6)	−0.010 (6)	0.000 (5)	0.053 (6)
C4	0.075 (8)	0.053 (6)	0.030 (5)	0.013 (6)	0.012 (5)	0.014 (4)
C5	0.038 (5)	0.075 (8)	0.046 (6)	−0.001 (5)	−0.006 (4)	0.034 (6)
N1	0.029 (3)	0.026 (4)	0.028 (3)	0.000 (3)	0.009 (3)	0.000 (3)
C6	0.043 (5)	0.040 (5)	0.037 (4)	0.007 (4)	0.002 (4)	−0.009 (4)
C7	0.058 (6)	0.048 (6)	0.043 (5)	−0.004 (5)	0.006 (5)	−0.018 (4)
C8	0.057 (6)	0.051 (6)	0.027 (4)	−0.017 (5)	0.007 (4)	−0.007 (4)
C9	0.044 (5)	0.051 (6)	0.028 (4)	−0.002 (5)	−0.004 (4)	0.000 (4)
C10	0.037 (5)	0.033 (5)	0.029 (4)	0.004 (4)	0.002 (3)	0.002 (3)
C11	0.035 (4)	0.046 (6)	0.023 (4)	0.004 (4)	−0.001 (3)	−0.004 (4)
O1	0.066 (5)	0.039 (4)	0.052 (4)	0.020 (4)	−0.001 (3)	−0.010 (3)
Sn1	0.0279 (3)	0.0395 (3)	0.0304 (3)	−0.0058 (3)	0.00236 (19)	0.0041 (3)
C12	0.061 (7)	0.095 (10)	0.056 (7)	−0.042 (7)	−0.004 (5)	−0.002 (7)
C13	0.107 (11)	0.073 (9)	0.067 (8)	−0.018 (8)	0.003 (7)	−0.019 (7)
C14	0.060 (7)	0.066 (8)	0.094 (9)	0.004 (6)	0.031 (6)	0.000 (7)
C15	0.066 (9)	0.121 (14)	0.148 (14)	0.026 (9)	0.051 (9)	0.016 (11)
C16	0.059 (7)	0.052 (7)	0.069 (7)	−0.015 (6)	0.000 (5)	0.029 (6)
C17	0.068 (8)	0.081 (9)	0.095 (9)	0.000 (7)	0.019 (7)	0.046 (8)

### Geometric parameters (Å, °)

**Table:**

Fe1—C11	1.728 (9)	C9—C10	1.379 (11)
Fe1—N1	1.984 (6)	C9—H9A	0.9500
Fe1—C5	2.080 (8)	C10—H10A	0.9500
Fe1—C4	2.082 (9)	C11—O1	1.159 (10)
Fe1—C2	2.096 (11)	Sn1—C16	2.166 (9)
Fe1—C1	2.103 (10)	Sn1—C14	2.174 (11)
Fe1—C3	2.104 (10)	Sn1—C12	2.177 (10)
Fe1—Sn1	2.5455 (13)	C12—C13	1.483 (16)
C1—C5	1.381 (16)	C12—H12A	0.9900
C1—C2	1.408 (19)	C12—H12B	0.9900
C1—H1A	0.9500	C13—H13A	0.9800
C2—C3	1.362 (19)	C13—H13B	0.9800
C2—H2A	0.9500	C13—H13C	0.9800
C3—C4	1.389 (15)	C14—C15	1.461 (17)
C3—H3A	0.9500	C14—H14A	0.9900
C4—C5	1.372 (14)	C14—H14B	0.9900
C4—H4A	0.9500	C15—H15A	0.9800
C5—H5A	0.9500	C15—H15B	0.9800
N1—C10	1.341 (10)	C15—H15C	0.9800
N1—C6	1.343 (10)	C16—C17	1.471 (14)
C6—C7	1.371 (12)	C16—H16A	0.9900
C6—H6A	0.9500	C16—H16B	0.9900
C7—C8	1.364 (13)	C17—H17A	0.9800
C7—H7A	0.9500	C17—H17B	0.9800
C8—C9	1.362 (13)	C17—H17C	0.9800
C8—H8A	0.9500		
			
C11—Fe1—N1	96.1 (3)	C6—N1—Fe1	121.9 (5)
C11—Fe1—C5	123.1 (4)	N1—C6—C7	124.0 (9)
N1—Fe1—C5	139.8 (4)	N1—C6—H6A	118.0
C11—Fe1—C4	95.2 (4)	C7—C6—H6A	118.0
N1—Fe1—C4	157.9 (4)	C8—C7—C6	118.9 (9)
C5—Fe1—C4	38.5 (4)	C8—C7—H7A	120.6
C11—Fe1—C2	136.0 (6)	C6—C7—H7A	120.6
N1—Fe1—C2	94.3 (4)	C9—C8—C7	118.8 (8)
C5—Fe1—C2	64.8 (5)	C9—C8—H8A	120.6
C4—Fe1—C2	64.6 (4)	C7—C8—H8A	120.6
C11—Fe1—C1	159.7 (4)	C8—C9—C10	119.2 (9)
N1—Fe1—C1	103.7 (4)	C8—C9—H9A	120.4
C5—Fe1—C1	38.5 (5)	C10—C9—H9A	120.4
C4—Fe1—C1	64.7 (4)	N1—C10—C9	123.5 (8)
C2—Fe1—C1	39.2 (5)	N1—C10—H10A	118.3
C11—Fe1—C3	102.0 (5)	C9—C10—H10A	118.3
N1—Fe1—C3	119.9 (4)	O1—C11—Fe1	178.3 (8)
C5—Fe1—C3	64.4 (4)	C16—Sn1—C14	105.3 (5)
C4—Fe1—C3	38.8 (4)	C16—Sn1—C12	105.9 (5)
C2—Fe1—C3	37.9 (5)	C14—Sn1—C12	105.5 (5)
C1—Fe1—C3	64.4 (5)	C16—Sn1—Fe1	111.9 (3)
C11—Fe1—Sn1	84.2 (3)	C14—Sn1—Fe1	112.1 (3)
N1—Fe1—Sn1	88.59 (19)	C12—Sn1—Fe1	115.3 (3)
C5—Fe1—Sn1	87.0 (3)	C13—C12—Sn1	117.3 (8)
C4—Fe1—Sn1	111.5 (3)	C13—C12—H12A	108.0
C2—Fe1—Sn1	138.8 (5)	Sn1—C12—H12A	108.0
C1—Fe1—Sn1	100.3 (4)	C13—C12—H12B	108.0
C3—Fe1—Sn1	149.5 (3)	Sn1—C12—H12B	108.0
C5—C1—C2	106.8 (11)	H12A—C12—H12B	107.2
C5—C1—Fe1	69.8 (6)	C12—C13—H13A	109.5
C2—C1—Fe1	70.1 (6)	C12—C13—H13B	109.5
C5—C1—H1A	126.6	H13A—C13—H13B	109.5
C2—C1—H1A	126.6	C12—C13—H13C	109.5
Fe1—C1—H1A	125.0	H13A—C13—H13C	109.5
C3—C2—C1	108.1 (11)	H13B—C13—H13C	109.5
C3—C2—Fe1	71.4 (7)	C15—C14—Sn1	117.2 (10)
C1—C2—Fe1	70.7 (6)	C15—C14—H14A	108.0
C3—C2—H2A	126.0	Sn1—C14—H14A	108.0
C1—C2—H2A	126.0	C15—C14—H14B	108.0
Fe1—C2—H2A	123.6	Sn1—C14—H14B	108.0
C2—C3—C4	108.5 (11)	H14A—C14—H14B	107.2
C2—C3—Fe1	70.8 (6)	C14—C15—H15A	109.5
C4—C3—Fe1	69.8 (6)	C14—C15—H15B	109.5
C2—C3—H3A	125.7	H15A—C15—H15B	109.5
C4—C3—H3A	125.7	C14—C15—H15C	109.5
Fe1—C3—H3A	125.3	H15A—C15—H15C	109.5
C5—C4—C3	107.7 (10)	H15B—C15—H15C	109.5
C5—C4—Fe1	70.7 (5)	C17—C16—Sn1	118.6 (8)
C3—C4—Fe1	71.5 (6)	C17—C16—H16A	107.7
C5—C4—H4A	126.2	Sn1—C16—H16A	107.7
C3—C4—H4A	126.2	C17—C16—H16B	107.7
Fe1—C4—H4A	123.4	Sn1—C16—H16B	107.7
C4—C5—C1	109.0 (10)	H16A—C16—H16B	107.1
C4—C5—Fe1	70.8 (5)	C16—C17—H17A	109.5
C1—C5—Fe1	71.6 (6)	C16—C17—H17B	109.5
C4—C5—H5A	125.5	H17A—C17—H17B	109.5
C1—C5—H5A	125.5	C16—C17—H17C	109.5
Fe1—C5—H5A	123.6	H17A—C17—H17C	109.5
C10—N1—C6	115.6 (7)	H17B—C17—H17C	109.5
C10—N1—Fe1	122.4 (5)		
			
C11—Fe1—C1—C5	−30 (2)	N1—Fe1—C5—C4	−144.9 (6)
N1—Fe1—C1—C5	162.9 (6)	C2—Fe1—C5—C4	−80.2 (8)
C4—Fe1—C1—C5	−37.2 (6)	C1—Fe1—C5—C4	−118.5 (10)
C2—Fe1—C1—C5	−117.3 (10)	C3—Fe1—C5—C4	−38.1 (7)
C3—Fe1—C1—C5	−80.3 (7)	Sn1—Fe1—C5—C4	130.9 (7)
Sn1—Fe1—C1—C5	71.8 (7)	C11—Fe1—C5—C1	168.2 (8)
C11—Fe1—C1—C2	87.8 (18)	N1—Fe1—C5—C1	−26.3 (10)
N1—Fe1—C1—C2	−79.8 (8)	C4—Fe1—C5—C1	118.5 (10)
C5—Fe1—C1—C2	117.3 (10)	C2—Fe1—C5—C1	38.3 (8)
C4—Fe1—C1—C2	80.1 (8)	C3—Fe1—C5—C1	80.4 (9)
C3—Fe1—C1—C2	37.0 (7)	Sn1—Fe1—C5—C1	−110.6 (8)
Sn1—Fe1—C1—C2	−170.8 (7)	C11—Fe1—N1—C10	−19.8 (7)
C5—C1—C2—C3	−1.4 (12)	C5—Fe1—N1—C10	172.4 (7)
Fe1—C1—C2—C3	−61.9 (8)	C4—Fe1—N1—C10	100.5 (11)
C5—C1—C2—Fe1	60.5 (7)	C2—Fe1—N1—C10	117.3 (8)
C11—Fe1—C2—C3	−32.4 (10)	C1—Fe1—N1—C10	155.9 (7)
N1—Fe1—C2—C3	−135.8 (7)	C3—Fe1—N1—C10	87.8 (8)
C5—Fe1—C2—C3	80.0 (7)	Sn1—Fe1—N1—C10	−103.8 (6)
C4—Fe1—C2—C3	37.3 (6)	C11—Fe1—N1—C6	162.7 (7)
C1—Fe1—C2—C3	117.7 (10)	C5—Fe1—N1—C6	−5.1 (9)
Sn1—Fe1—C2—C3	131.4 (7)	C4—Fe1—N1—C6	−77.0 (11)
C11—Fe1—C2—C1	−150.1 (8)	C2—Fe1—N1—C6	−60.2 (8)
N1—Fe1—C2—C1	106.5 (8)	C1—Fe1—N1—C6	−21.6 (8)
C5—Fe1—C2—C1	−37.7 (7)	C3—Fe1—N1—C6	−89.7 (8)
C4—Fe1—C2—C1	−80.4 (7)	Sn1—Fe1—N1—C6	78.7 (6)
C3—Fe1—C2—C1	−117.7 (10)	C10—N1—C6—C7	0.0 (13)
Sn1—Fe1—C2—C1	13.8 (10)	Fe1—N1—C6—C7	177.7 (7)
C1—C2—C3—C4	1.7 (12)	N1—C6—C7—C8	−0.4 (15)
Fe1—C2—C3—C4	−59.8 (7)	C6—C7—C8—C9	0.4 (15)
C1—C2—C3—Fe1	61.5 (7)	C7—C8—C9—C10	0.0 (14)
C11—Fe1—C3—C2	157.6 (8)	C6—N1—C10—C9	0.4 (12)
N1—Fe1—C3—C2	53.3 (8)	Fe1—N1—C10—C9	−177.3 (7)
C5—Fe1—C3—C2	−81.3 (8)	C8—C9—C10—N1	−0.4 (14)
C4—Fe1—C3—C2	−119.1 (11)	C11—Fe1—Sn1—C16	−42.6 (4)
C1—Fe1—C3—C2	−38.3 (7)	N1—Fe1—Sn1—C16	53.7 (4)
Sn1—Fe1—C3—C2	−103.3 (11)	C5—Fe1—Sn1—C16	−166.2 (5)
C11—Fe1—C3—C4	−83.3 (7)	C4—Fe1—Sn1—C16	−135.9 (5)
N1—Fe1—C3—C4	172.4 (6)	C2—Fe1—Sn1—C16	148.6 (6)
C5—Fe1—C3—C4	37.9 (7)	C1—Fe1—Sn1—C16	157.4 (5)
C2—Fe1—C3—C4	119.1 (11)	C3—Fe1—Sn1—C16	−146.4 (9)
C1—Fe1—C3—C4	80.8 (8)	C11—Fe1—Sn1—C14	−160.6 (5)
Sn1—Fe1—C3—C4	15.8 (13)	N1—Fe1—Sn1—C14	−64.4 (4)
C2—C3—C4—C5	−1.3 (11)	C5—Fe1—Sn1—C14	75.7 (5)
Fe1—C3—C4—C5	−61.8 (7)	C4—Fe1—Sn1—C14	106.1 (5)
C2—C3—C4—Fe1	60.4 (7)	C2—Fe1—Sn1—C14	30.5 (6)
C11—Fe1—C4—C5	−140.1 (7)	C1—Fe1—Sn1—C14	39.3 (5)
N1—Fe1—C4—C5	99.5 (11)	C3—Fe1—Sn1—C14	95.5 (9)
C2—Fe1—C4—C5	80.8 (8)	C11—Fe1—Sn1—C12	78.7 (5)
C1—Fe1—C4—C5	37.3 (7)	N1—Fe1—Sn1—C12	174.9 (5)
C3—Fe1—C4—C5	117.2 (10)	C5—Fe1—Sn1—C12	−45.0 (6)
Sn1—Fe1—C4—C5	−54.3 (7)	C4—Fe1—Sn1—C12	−14.6 (5)
C11—Fe1—C4—C3	102.7 (8)	C2—Fe1—Sn1—C12	−90.2 (7)
N1—Fe1—C4—C3	−17.7 (14)	C1—Fe1—Sn1—C12	−81.4 (6)
C5—Fe1—C4—C3	−117.2 (10)	C3—Fe1—Sn1—C12	−25.2 (9)
C2—Fe1—C4—C3	−36.4 (8)	C16—Sn1—C12—C13	89.4 (10)
C1—Fe1—C4—C3	−80.0 (9)	C14—Sn1—C12—C13	−159.2 (10)
Sn1—Fe1—C4—C3	−171.5 (7)	Fe1—Sn1—C12—C13	−35.0 (11)
C3—C4—C5—C1	0.4 (11)	C16—Sn1—C14—C15	64.0 (12)
Fe1—C4—C5—C1	−61.8 (7)	C12—Sn1—C14—C15	−47.8 (12)
C3—C4—C5—Fe1	62.3 (7)	Fe1—Sn1—C14—C15	−174.1 (10)
C2—C1—C5—C4	0.6 (11)	C14—Sn1—C16—C17	−65.9 (11)
Fe1—C1—C5—C4	61.3 (7)	C12—Sn1—C16—C17	45.6 (11)
C2—C1—C5—Fe1	−60.7 (7)	Fe1—Sn1—C16—C17	172.0 (9)
C11—Fe1—C5—C4	49.7 (8)		
